# A Compact Magnetic Field-Based Obstacle Detection and Avoidance System for Miniature Spherical Robots

**DOI:** 10.3390/s17061231

**Published:** 2017-05-28

**Authors:** Fang Wu, Akash Vibhute, Gim Song Soh, Kristin L. Wood, Shaohui Foong

**Affiliations:** Engineering Product Development, Singapore University of Technology and Design, 8 Somapah Road, Singapore 487372, Singapore; fang_wu@sutd.edu.sg (F.W.); akash.roboticist@gmail.com (A.V.); sohgimsong@sutd.edu.sg (G.S.S.); kristinwood@sutd.edu.sg (K.L.W.)

**Keywords:** obstacle detection, obstacle avoidance, spherical robot, magnet assembly, miniature robot, sensor optimization

## Abstract

Due to their efficient locomotion and natural tolerance to hazardous environments, spherical robots have wide applications in security surveillance, exploration of unknown territory and emergency response. Numerous studies have been conducted on the driving mechanism, motion planning and trajectory tracking methods of spherical robots, yet very limited studies have been conducted regarding the obstacle avoidance capability of spherical robots. Most of the existing spherical robots rely on the “hit and run” technique, which has been argued to be a reasonable strategy because spherical robots have an inherent ability to recover from collisions. Without protruding components, they will not become stuck and can simply roll back after running into bstacles. However, for small scale spherical robots that contain sensitive surveillance sensors and cannot afford to utilize heavy protective shells, the absence of obstacle avoidance solutions would leave the robot at the mercy of potentially dangerous obstacles. In this paper, a compact magnetic field-based obstacle detection and avoidance system has been developed for miniature spherical robots. It utilizes a passive magnetic field so that the system is both compact and power efficient. The proposed system can detect not only the presence, but also the approaching direction of a ferromagnetic obstacle, therefore, an intelligent avoidance behavior can be generated by adapting the trajectory tracking method with the detection information. Design optimization is conducted to enhance the obstacle detection performance and detailed avoidance strategies are devised. Experimental results are also presented for validation purposes.

## 1. Introduction

**Figure 1:**
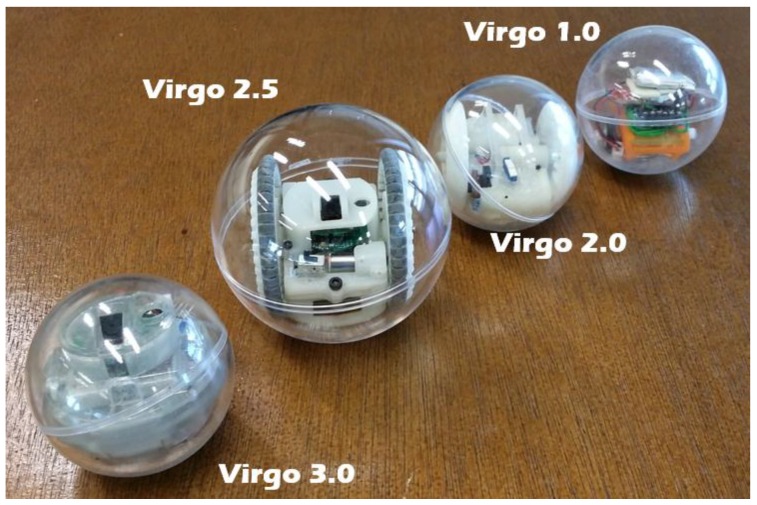
VIRGO, a spherical robot developed in SUTD [17].

Over the past decades, autonomous mobile robots [1,2,3,4] have received significant attention in the robotics community and challenges related to their navigation [5,6,7] have motivated countless studies. Nonholonomic robots [8,9], being one of the vastly developed categories, have wide applications in surveillance and transportation. Various locomotion mechanisms [10,11,12,13] have been employed depending on the application environment. Spherical robots, due to their natural geometry and efficient locomotion technique, have remained a promising research area. As a closed system, all the functional components are sealed inside the shell and protected from potential external hazards or interference, which makes spherical robots especially suitable for reconnaissance, rescue missions and even underwater operations [14,15,16]. VIRGO [17,18] is a novel miniature spherical robot developed in Singapore University of Technology and Design (SUTD) with unique surveillance capabilities and high battery endurance. The drivetrain consists of a differential drive cart unit and operates with a mechanism similar to that of a hypocyclic geartrain. This driving mechanism has been chosen due to its simplicity and potential to miniaturize. Consistent efforts have been made to improve its dynamic performance while shrinking the overall size. Currently it is capable of achieving a 3 Wh/113.1 cm^3^ energy density and 200 mW operational power draw. The radius of the latest version is 3 cm, as shown in Figure 1.

With the limited space and computational power, the onboard sensing hardware and algorithms need to be carefully designed and developed. Navigation of mobile robots have usually been discussed in three different directions: motion planning, trajectory tracking and obstacle avoidance. Global motion planning methods including potential field methods [19] and sampling based planners [20,21] have been extensively studied. These algorithms typically rely on mathematical analysis of determined geometries and require complete information of the operation space and obstacles in prior. In order to develop a decentralized robotic system while considering the size constraint and limited computation capability on board, no prior or centralized motion planning has been pursued for VIRGO. Currently the trajectory tracking and point-to-point locomotion of VIRGO is handled by a Pure-Pursuit controller. As described in [17], pure-pursuit algorithm computes the curvature of the ongoing path which starts from the robot current location and ends at the target waypoint. The performance of the waypoint tracking has been validated in previous works. Regarding the obstacle avoidance, it has been repeatedly argued that one of the natural benefits of a spherical robot is its capability to recover from collisions. And for that specific reason, there are very limited studies on the obstacle avoidance for spherical robots. However, for decentralized yet miniature spherical robots like VIRGO, obstacle avoidance capability is necessary not only to optimize its navigation efficiency, but also to improve the durability of the robot without sacrificing its weight. 

Commonly used proximity detection solutions include ultrasonic transceivers, infrared and optical sensors, wireless sensor network, and electromagnetic devices such as inductive/capacitive sensors. Ultrasonic transceivers [22,23,24], although an economical and robust choice for obstacle detection, cannot be used for spherical robots due to the plastic shell that completely encloses the drive cart unit. As a result, the ultrasound signal cannot penetrate the shell. Cameras and infrared sensors are widely available on the market and have been used for navigation purposes [25,26,27], however, their application requires a supporting pattern recognition algorithm, which would demand considerable computational efforts and high processing capability. Also, their performance could soon deteriorate as the shell transparency quickly reduces due to abrasion. Wireless sensor networks [28] usually drain the power fast and many of them, such as UWB technique [29], require external beacons to be set up before the robot operation. Commercial inductive and capacitive sensors [30], although not affected by the shell at all, are simply too big to fit in the sphere. Therefore, a passive magnetic sensor [18] has been proposed and directional proximity detection has been achieved by using a magnet assembly to magnify the magnetic disturbance due to approaching ferromagnetic obstacles. The only sensor needed is the magnetometer, which adds minimum energy consumption and can even be neglected when using the existing magnetometer inside the inertial measurement unit (IMU). 

In this paper, a magnetic proximity sensor is optimized and integrated with the robot configuration to achieve a reasonable detection range without affecting the robot behavior. The pure pursuit controller is adapted to handle the obstacle avoidance action by only employing data from the proximity sensor. Practical issues when applying the proximity sensor are detailed and analyzed. The experimental performance is finally verified against the ground truth using an optical motion capture system.

## 2. Passive Magnetic-Based Proximity Sensor 

### 2.1. Design Concept and Modeling

**Figure 2:**
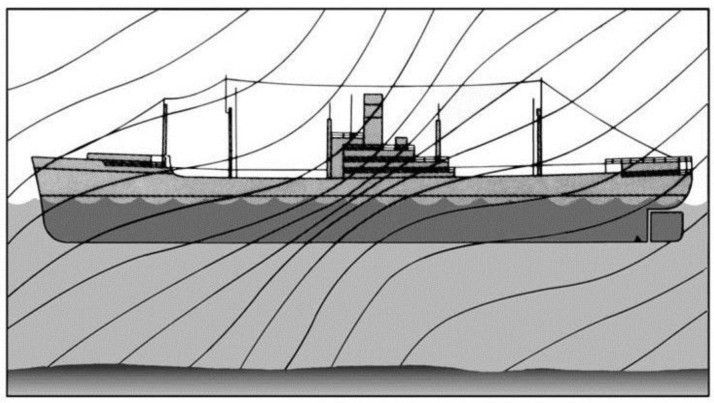
The distorted field due to a steel vessel when the earth magnetic field is present [34].

Existing researches have frequently exploited the Earth’s magnetic field to detect the magnetic anomalies [31,32,33]. Modelling the Earth as a giant dipole magnet, the Earth’s magnetic field on the ground can be approximated to be uniform and parallel to the ground. This field requires no external source and distorts wherever a ferromagnetic object is presented. Figure 2 [34] shows the distortion of Earth’s magnetic field in the vicinity of a steel vessel and the line density represents the magnetic field strength. 

To apply this method in everyday usage, one of the main difficulties is the weakness of the disturbance field and the corresponding low signal/noise ratio. With the Earth’s magnetic field strength ranging from 25 to 65 microteslas, the distortion due to an object significantly smaller than a steel vessel can be too small to detect reliably, even by modern sensors. What’s more, the distortion itself is directional and also depends on the direction of the Earth’s magnetic field as well as the geometry of the ferromagnetic object. When only the Earth’s magnetic field is present, it’s often difficult to model the distortion field. While material and size of the object have a proportional relationship with the magnitude of the distortion field and therefore can be normalized, the magnetic orientation of the object relative to the geomagnetic field remains unknown because the geomagnetic field direction differs from location to location. Various methods have been used to magnify and abstract information from the small distortion field. Some of them sacrificed the directional feature to achieve a usable signal. Here, a sensor structure is proposed that features a specially designed passive magnetic field to amplify the distortion field. Because of the specific and unique field design, it is possible to compute the magnetic orientation of this object. And this capability will permit directional detection, which is paramount for path planning purpose in robotic applications.

Taking inspiration from the electrical Wheatstone bridge which allows small resistive changes to be detected through hardware amplification, the sensing system developed here consists of a balanced magnetic flux loop. With intelligent placement of two permanent magnets, the key idea is to artificially create a magnetic field that possesses a very high spatial gradient. When a ferromagnetic object is in the vicinity, it disrupts the magnetic field. Since the magnetic fluxes are continuous, an instantaneous field distortion can be detected using a low-powered magnetic field sensor. This distortion field is much stronger than the case when only the Earth magnetic field is present, as demonstrated in previous work [18]. Considering the compact robot and obstacle size used in this work, it is reasonable to neglect the distortion due to the earth magnetic field here.

**Figure 3:**
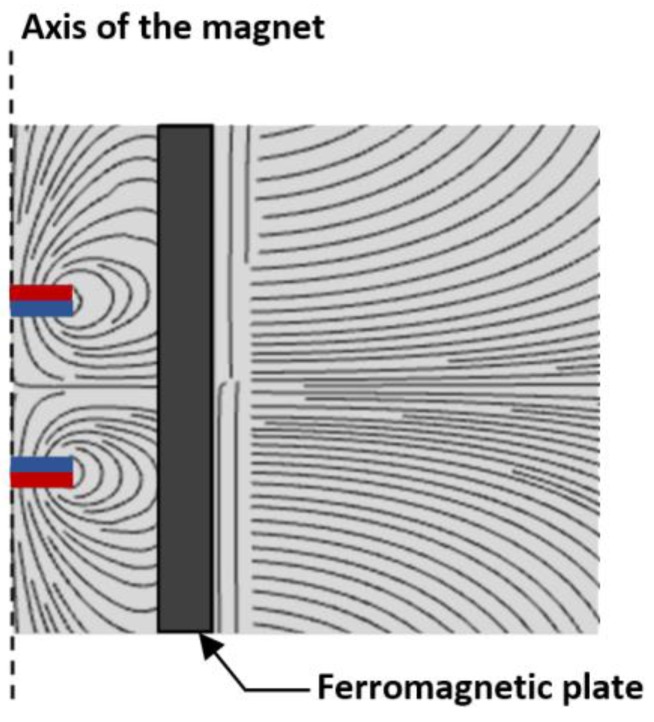
Distorted field pattern due to an approaching object.

**Figure 4:**
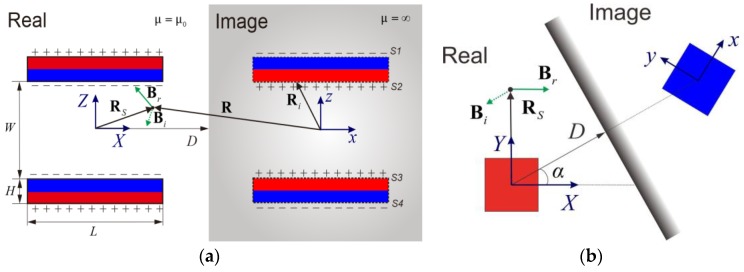
Modeling of the approaching obstacle as image charges: (**a**) approaching angle *α* = 0; (**b**) approaching angle *α* ≠ 0.

The magnetic field lines from two opposing permanent magnets are depicted in Figure 3. Due to high permeability of the ferromagnetic material, magnetic field lines distort around the ferromagnetic plate so that the horizontal component of the magnetic field reduces to almost zero near the boundary. The model of the distortion field has been detailed in previous work [18] and a 2D model of the distorted magnetic field is shown in Figure 4. The magnetic charge model approximates the volume of magnetic sources by utilizing a series of positive and negative charges. Positive charges are located on the north pole of the magnet and negative charges on the south pole. When an object of infinite magnetic permeability and height approaches the permanent magnets as in Figure 4, the resultant field can be calculated by employing the image method where image charges with opposite signs are placed inside the object. Then the magnetic field outside the object can be accurately represented by summing up the contributions from the real charges and the image charges as **B***_r_* + **B***_i_*. The distortion field due to the object can be simply calculated as the contribution from the image charges only. As depicted in Figure 4, the magnetic field from the image charges can be first calculated in the image coordinate system *x-y-z* and then transformed into the real coordinate system *X-Y-Z*. The geometric relation between these two coordinate systems is dependent on the distance *D* to the object and its approaching angle *α* in the real coordinate system. The closed-form solution can be expressed as (1).

(1) $$\Delta B=B_{i}=T\times \frac{\mu_{0}}{4\pi }\left(\oint_{S1+S4}\frac{-\sigma_{i}(R-R_{i})}{{\left|R-R_{i}\right|}^{3}}dS+\oint_{S2+S3}\frac{\sigma_{i}(R-R_{i})}{{\left|R-R_{i}\right|}^{3}}dS\right)$$

where **T** represents the transformation matrix from the image coordinate system *x-y-z* to the real coordinate system *X-Y-Z*; S1, S2, S3 and S4 refers to the surface of magnetic poles; $\sigma_{i}$ represents the image charge, which is a constant value when assuming the magnetization of the magnet is constant; **R** − **R***_i_* represents the vector from the location of the image charges to the location of calculated magnetic field in the *x-y-z* coordinate system. 

Equation (1) can be simplified when the sensing system is located at the origin of the real coordinate system, and the detailed derivation is included as Appendix A. The approaching angle can be easily computed from the normalized field variation as below:

(2) $$\alpha =\arctan(\frac{\Delta B_{y}}{\Delta B_{x}})\;\mathrm{when}\;R_{S}={\left[\begin{array}{ccc} 0 & 0 & 0 \end{array}\right]}^{T}$$

This equation implies that when the sensor is placed at the central point between the two magnets, the distortion field due to a ferromagnetic object will simply point to the object location. Although the derivations above are done under the assumption that the object is infinitely large and with infinite permeability, the conclusion would still be true for finite objects since the VIRGO robot is significantly smaller than most obstacles. In addition, the modeling analysis here reflects the distorted field pattern, not the exact field strength. It is expected that the distortion field magnitude would decrease while the normalized vector remains to be as (2). Experimental demonstration has been presented in previous work [18]. 

### 2.2. Design Integration and Optimization

**Figure 5:**
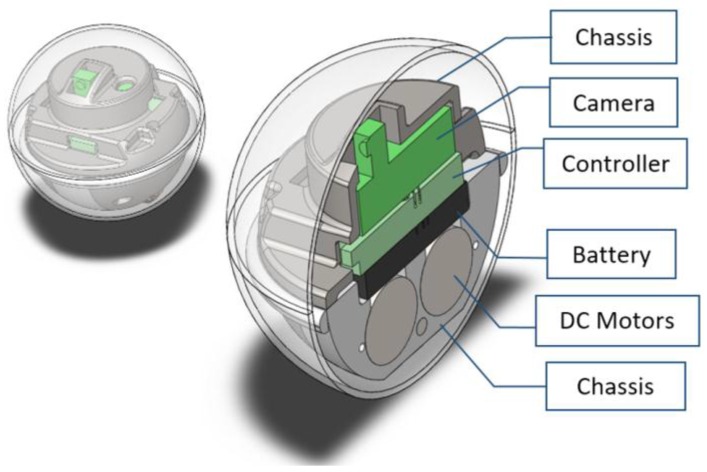
Configuration of VIRGO.

To adapt the magnetic proximity sensor to the spherical robot, the configuration of VIRGO is examined first. As shown in Figure 5, all the functional components including the camera, controller, battery and the DC motors are fitted inside a chassis that located at the central area inside the sphere. Two wheels are installed at the bottom of the chassis and transfer the motion to the shell. 

Two permanent magnets with opposing magnetization can be placed at the top and bottom of the moving chassis, therefore seamless integrated into the robot with minimum re-configuration. With the camera facing front and constraining the diameter of the magnet, magnets of square shape are chosen over the disk shape in order to achieve maximum area. The existing IMU has an embedded magnetometer and can be used for this sensing purpose. Here an extra magnetometer is used so that the obstacle detection and robot motion performance can be examined separately.

In previous section, a closed form solution as (1) has been presented to compute the distortion field due to an approaching obstacle. And the magnitude of the distortion field can be expressed as (3).

(3) $$\left|\Delta B\right|=\frac{\mu_{0}\sigma_{i}\left|R\right|}{4\pi }(\oint_{S2+S3}\frac{1}{{\left|R-R_{i}\right|}^{3}}dS-\oint_{S1+S4}\frac{1}{{\left|R-R_{i}\right|}^{3}}dS)$$

where **R** and **R***_i_* represent the location of the sensor and image charges in the image coordinate system; *S*1, *S*2, *S*3 and *S*4 refer to the pole areas of the image magnets. By observing the equation, it is apparent that the magnitude decreases as the distance to the obstacle increases. Equation (3) cannot be used to accurately estimate the actual magnitude of distortion field in real life due to the infinite assumptions on the magnetic permeability of the obstacle and the obstacle height. With finite objects of finite magnetic permeability, the distortion of the magnetic field is expected to happen on a smaller scale and within a smaller area. However, both the size and geometric configurations of the magnets are considered in the model and their effects on the sensing performance can be reasonably studied. 

**Figure 6:**
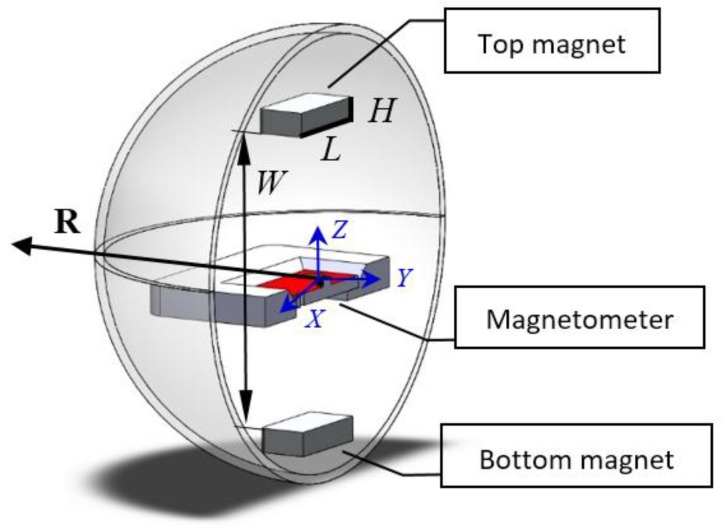
Geometric parameters of the magnetic proximity sensor.

As shown in Figure 6, the length and height of the square magnets are defined as *L* and *H*. And the separation distance between two magnets is defined as *W*. Although these are the explicit geometric parameters, they are not suitable as the direct inputs for optimization purpose. 

To understand these variables, one should first be aware that once the material of a magnet is set, strength of the magnet is decided by its overall size. And its shape decides the pattern of the magnetic field distribution, which means the magnet length and height affect its magnetic field in a correlated way, rather than independently. Therefore, the aspect ratio of the magnet is defined as γ=L/H and used as the first optimization variable. The magnet volume, which accounts for its general strength, can be calculated as *V* = *HL*^2^ and used as the second optimization variable. The separation distance *W* between two magnets is the third optimization variable. To maximize the magnitude of the distortion field, the optimization problem can be formed as (4):

(4) $$(V,\gamma ,W)=\mathrm{argmax}\sum_{R=R_{1},\ldots R_{N}}\left|\Delta B\right|\left|R\right|$$

where the magnetic field locations R_1_, …, R_N_ are a series of points along the radial direction in the *XY* plane as in Figure 6. The absolute values of R are used as weighting factors here because more emphasis should be given to the locations farther away from the robot for obstacle detection purpose.

Simulation studies of the geometric variables are detailed in Section 3 and optimization procedures are also presented.

### 2.3. Detection and Avoidance Strategies

The mechanical integration and configuration optimization of the obstacle detection system have been discussed. By continuously monitoring the magnetic field measurements and interpreting the disturbance, the approaching of a ferromagnetic obstacle can be detected. In comparison to a standalone sensor on the ground [18], a detection system on a moving platform needs to update the reference field continuously so that the distortion field due to an approaching obstacle can be distinguished from the noisy measurements due to changing locations. A moving-window average of measurements is computed continuously and the newest results are used as the reference field. And by subtracting this reference data from every new measurement, the calibrated data is obtained and used for obstacle detection purpose. 

**Figure 7:**
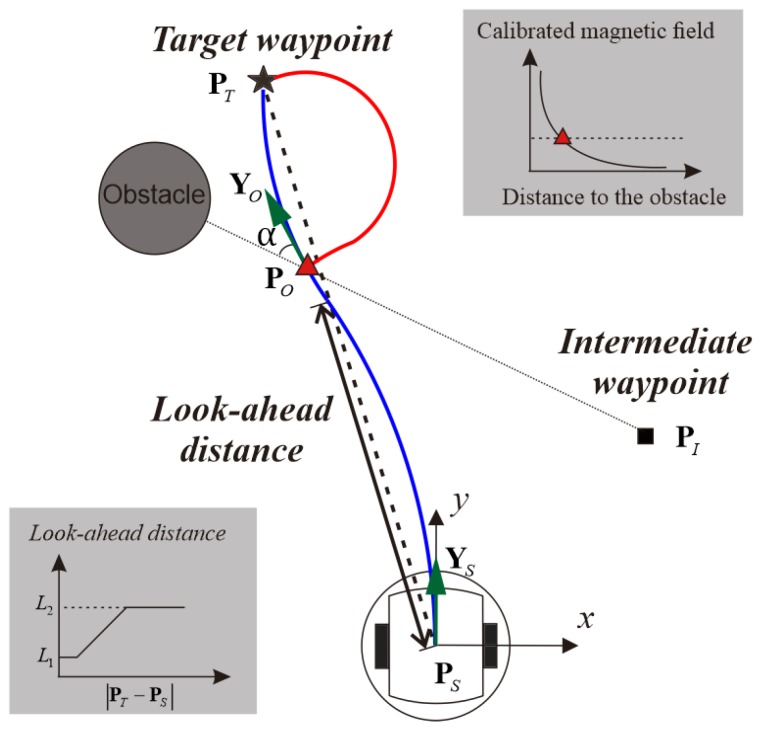
Illustration of the pure-pursuit method and obstacle avoidance behavior integration.

Currently pure-pursuit algorithm has been implemented on the VIRGO platform for trajectory tracking. When a target waypoint **P***_T_* is given, the robot controller calculates the distance between its current location **P***_S_* and the given waypoint. If the distance is within the predefined range of look-ahead distance, the pure-pursuit controller calculated a trajectory based on the distance and the robot heading **Y***_S_*. And the wheel speed can be decided accordingly. If the distance is smaller than the minimum look-ahead distance *L*_1_, the trajectory is generated based on the minimum look-ahead distance *L*_1_ and the robot heading **Y***_S_*. *L*_1_ is defined as twice the diameter of the robot so that the robot doesn’t attempt to reach an impossible location. If the distance is bigger than the maximum look-ahead distance *L*_2_, as shown in Figure 7, the controller generates a trajectory based on the maximum look-ahead distance and the robot heading **Y***_S_*. *L*_2_ serves as a tuning coefficient to adjust the robot motion behavior. With a big value for *L*_2_, the robot chooses to move in a huge curve when the target waypoint is on the side; with a small value, the robot would move in a meandering path. The executed trajectory consists of multiple curves, as depicted below using blue lines. To prevent the motion deviation from accumulating, the robot controller computes the curvature iteratively instead of once and for all. During this process, if the robot is following the computed trajectory accurately, the same trajectory will repeatedly be generated until the full course is completed. If the robot drift away from the computed trajectory, a new path will be generated to correct the error. 

Once an obstacle is detected, an avoidance action need to be generated to adjust the robot course. Given the limited computation capability and available time for the robot to plan an action, techniques based on complex geometry [35] or heavy computation [36] are not suitable here. To integrate the obstacle avoidance action into the pure pursuit controller, new intermediate waypoints are added into the closed loop once the obstacle is detected. Due to the specific scheme of pure-pursuit algorithm, the robot would inevitably move forward regardless of the waypoints. To prevent collision, when the obstacle is right in front of the robot, the robot is given a waypoint of its current location **P***_O_* and then perform a turn on the spot. The turning direction could be clockwise or counterclockwise depending on the obstacle approaching angle α. Due to the uncertainty of the obstacle size, the turning angle is set to be 90° so that the avoidance behavior has a higher success rate. And then, an intermediate waypoint **P***_I_* can be computed from the sensor reading, current location **P***_O_* and robot heading **Y***_O_* as (5). As depicted with a red line in Figure 7, the robot can avoid the obstacle automatically by following the trajectory towards the newly added waypoint and then proceeding to the target waypoint:

(5) $$P_{I}=P_{O}-L_{1}\left[\begin{array}{cc} \cos\alpha & -\sin\alpha \\ \sin\alpha & \cos\alpha \end{array}\right]Y_{O}$$

**Figure 8:**
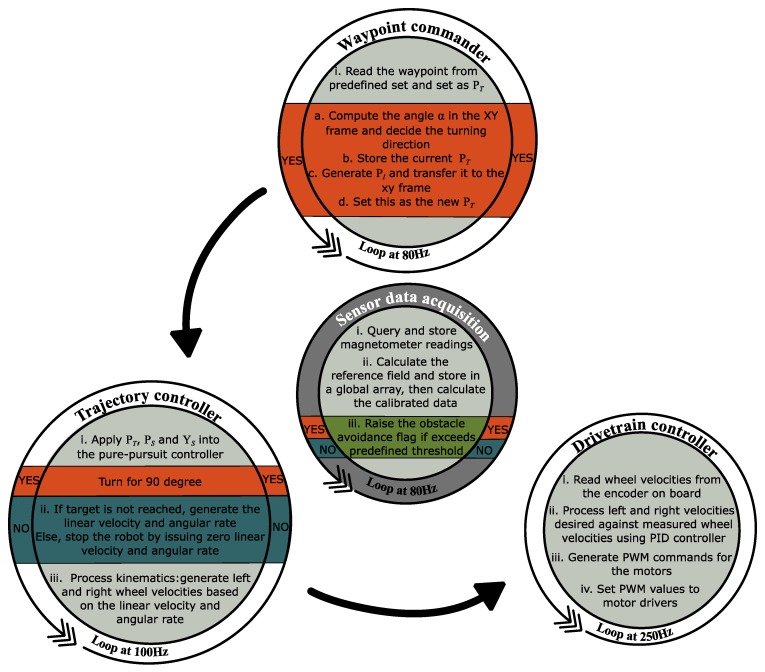
Illustration of the detailed algorithm.

Once a decision of obstacle being detected has been made, the control unit will trigger an interruptive protocol, which includes stopping the motor, turning on the spot and going to the intermediate waypoint. After that, the previous waypoint will be retrieved and the trajectory is recalculated from the current location. If the robot speed is beyond the stability threshold, which means an abrupt stop will cause the robot to tumble, the robot will slow down first and then stop entirely. This choice will be made by the control unit depending on the preset stability threshold and the robot proceeding speed when detecting the obstacle. As illustrated in Figure 8, the detailed algorithm includes four interactive threads: sensor data acquisition, waypoint commander, trajectory controller, and drivetrain controller. The sensor data acquisition thread processes the measurements and decides the state of obstacle avoidance flag. The waypoint commander produces the target waypoint from a predefined set depending on the obstacle avoidance flag. The trajectory controller computes the wheel velocities from the target waypoint depending on the obstacle avoidance flag. And at last, the drivetrain controller commands the motor with the computed wheel velocities. It is worth noting that although certain variables such as target waypoint, obstacle avoidance flag and wheel velocities are passed between different threads, there is no chronology order in the execution of all four threads. They are being initiated and executed all at once with different loop frequencies. 

## 3. Simulation Studies of the Optimization Process

It is worth mentioning that the geometry of a magnet is constrained by commercial standards and cannot be any random values. It is against the intention of designing a simple and compact proximity sensor if a magnet of special geometry must be used. Considering the overall configuration of the robot in Figure 6, magnets of different geometries as in Table 1 are selected for the comparison studies. Magnet *a* and *b* have the same volume but different aspect ratios, while magnet *c* and *d* have the same doubled volume and different aspect ratios. Magnetic field from each of these magnets is computed from the magnetic charge model outlined in the Appendix A.

### 3.1. Analysis on the Effect of Magnet Geometry

**Figure 9:**
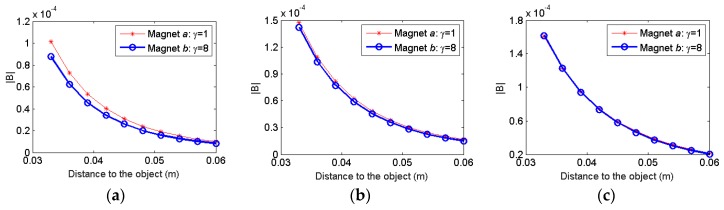
Comparison of the distortion field when magnets with different aspect ratios are used: (**a**) *W* = 2 cm; (**b**) *W* = 4 cm; (**c**) *W* = 6 cm.

To study the effect of aspect ratio, magnet *a* and *b* are first used with different separation distances (*W*). The magnitude of the distortion magnetic field is simulated as Figure 9. It is observed that when the distance between the two magnets is 2 cm, the magnitude of the distortion field increases as the aspect ratio decreases. Proximity sensor using the magnet *b* generates a bigger disturbance field than magnet *a*. The difference is 13.66% between the magnet *a* and magnet *b*. As the distance between the two magnets increase, the effect of the aspect ratio is weakened. The difference between the magnet *a* and magnet *b* is only 3.8% when the distance between the magnets is 4 cm, and further reduced to 0.68% when the distance between the magnets is 6 cm. This comparison indicates that although the aspect ratio of the magnet does have an impact on the sensor performance, it might not be the dominant one depending on the distance between the magnets.

**Figure 10:**
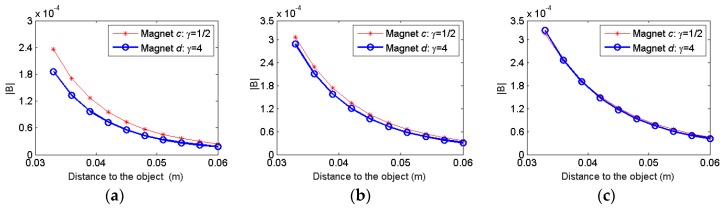
Comparison of the distortion field when magnets with doubled volume and different aspect ratios are used: (**a**) *W* = 2 cm; (**b**) *W* = 4 cm; (**c**) *W* = 6 cm.

To study the effect of the magnet volume, magnet *c* and magnet *d* are used in simulation and the distortion magnetic field is computed as Figure 10. It is seen that the magnitude of the distortion field largely increases with the magnet volume regardless of the various distances between the two magnets. And even though the aspect ratio of these two magnets are different with the previous three magnets, the same tendency is observed that the effect of the aspect ratio is diminished as the separation distance between the two magnets increases.

### 3.2. Analysis on the Effect of Separation Distance

Compared to the aspect ratio, the volume of the magnet has a more dominant influence on the detection performance, especially when the distance between the two magnets is bigger. Here, to study the effect of separation distance (*W*) between the two magnets, the geometries of magnet *c* and *d* are used. They have the similar volume but different aspect ratios. A summation of the magnitude of the distortion fields is computed when the obstacle is located at a series of incremental locations. 

**Figure 11:**
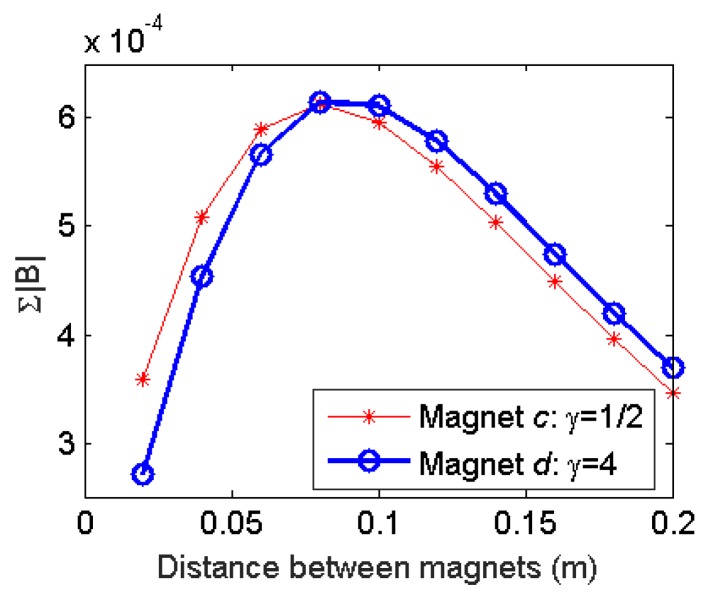
Effect of the distance between two magnets on the distortion field strength.

Although magnet *c* and *d* have different aspect ratios, similar conclusions can be drawn about the relation between the distortion field strength and the distance between the magnets. As shown in Figure 11, the magnitude of the distortion field increases with the distance between two magnets and reaches the maximum value when distance between the magnets is 8 cm. This is a theoretical optimal distance and can be used as guidelines when the actual distance is decided. 

### 3.3. Optimization of Integration into Spherical Robot

**Figure 12:**
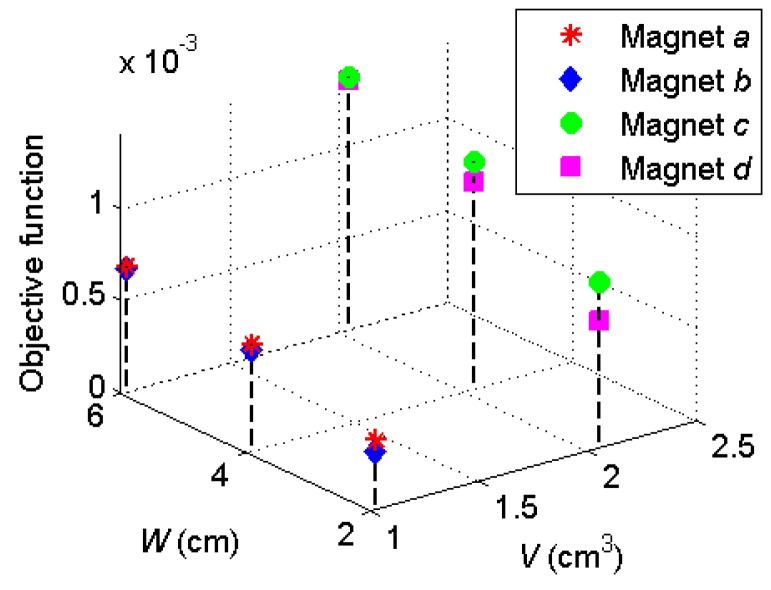
Comparison of the objective functions for different magnets.

In summary, three optimization variables have been discussed: aspect ratio of the permanent magnet, volume of the permanent magnet, and the separation distance between the two magnets. The design optimization strategies for an onboard magnetic proximity sensor can be interpreted. First, the volume of the permanent magnet has a proportional relation with the distortion field strength, which means a bigger magnet is always a better option if the space and load demands are met. Second, after fixing the volume of the magnet, a random aspect ratio can be decided. Then the distance between the two magnets can be decided considering the theoretical optimal distance and the spatial configuration inside the robot. Finally, if a large distance between the magnets has been chosen, the aspect ratio of the magnet has little influence over the distortion field strength, therefore can be decided only from the mechanical design point of view. If a small distance between the magnets has been decided, magnets with smaller aspect ratio should be used. Considering the four magnets in Table 1, the objective function in (4) can be calculated and plotted in Figure 12. 

The optimal combination is magnet *c* or *d* with 6 cm separation distance. Since magnet *c* has a height of 1.27 cm and will conflict with the central functional components in Figure 5, magnet *d* is used in the experiments below. 

## 4. Experimental Results

**Figure 13:**
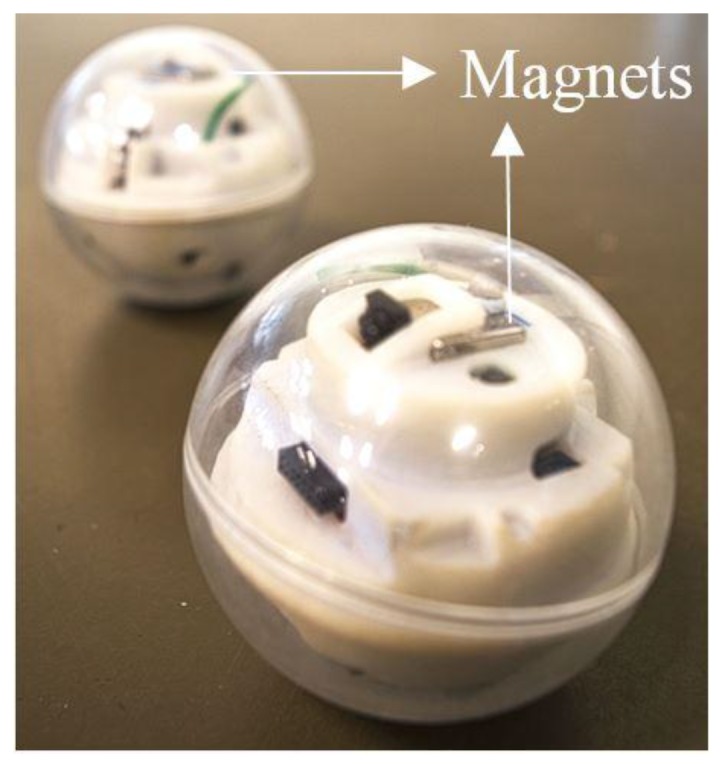
VIRGO robot integrated with the embedded magnetic proximity sensor.

**Figure 14:**
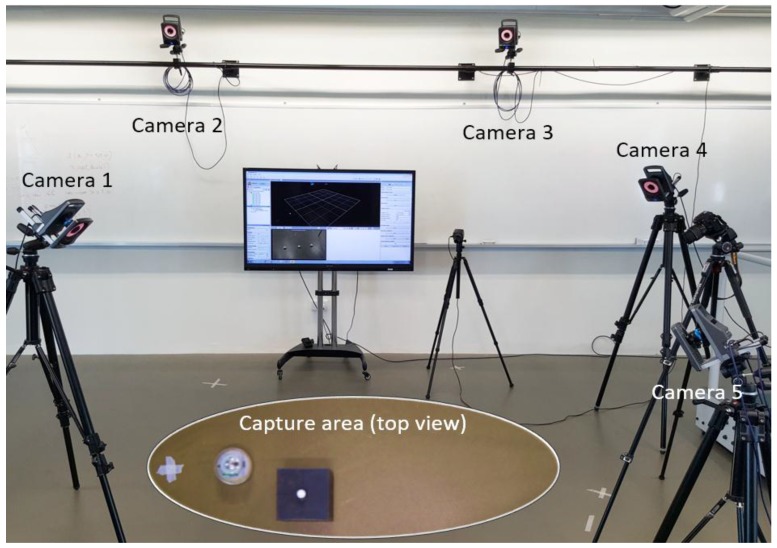
Experimental setup in the VICON system.

Given the robot size and configuration shown in Figure 5 and considering the design optimization strategies detailed in previous section, two magnets of size (*L* = 1/2 inch, *H* = 1/8 inch) are chosen and installed on the robot platform, as shown in Figure 13. The mechanical parameters of the robot are detailed in Table 2. To evaluate the obstacle avoidance performance, an optical motion tracking system (T160, VICON, Oxford, UK) consisting of five IR cameras is used to capture the robot behavior, as shown in Figure 14.

### 4.1. Calibration

To validate the practical sensor performance and calibrate the angle detection accuracy, a VS068 industrial robot (DENSO, Kariya, Japan) is used to move a ferromagnetic object around the sensor. The robot is fixed on the table of nonmagnetic material and a steel cube with 8 cm length is moved by the robot hand. A MAG3110 three-axis magnetometer (Freescale, Austin, TX, USA) is installed between the two magnets and an Arduino Uno board (Arduino, Somerville, MA, USA) is used for data acquisition purpose.

**Figure 15:**
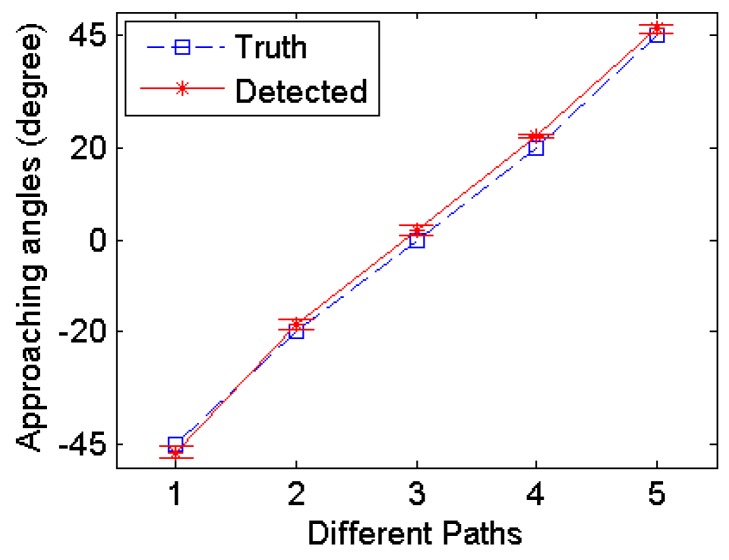
Angle detection accuracy along different approaching paths.

Since the robot will only run into obstacles in front of it, the object is commanded to approach the robot from five different angles in the frontal range and the approaching angle of the object can be calculated from the magnetic field based on (2). For each approaching angle, an average value is computed among different distances to the object, as shown in Figure 15. And the variations of the detected angle due to difference distances are shown using error bars above and below the average value. The average detection errors and maximum detection errors are detailed in Table 3. A maximum error of 3.2° happens when the actual approach angle is 0°. Considering the 90-degree calibration range, the angle detection errors range from 1.4 to 3.5%. No apparent relation between the angle detection error and the approaching angles or the distance to the object have been observed. Expected reasons for the angle detection errors include the imperfect magnetic field distribution from the permanent magnet and small misalignments between the two magnets. 

**Figure 16:**
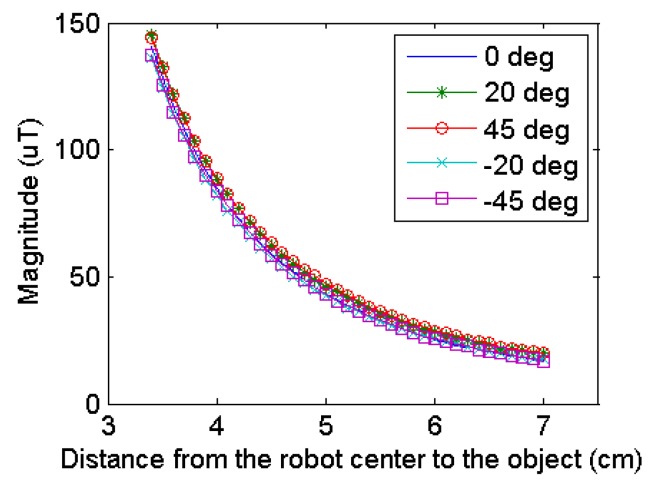
Relation between the measured field and the distance to the ferromagnetic object.

The magnitude of the measured distortion field is shown in Figure 16. It can be seen the maximum distortion field is detected when the object is 3.5 cm away from the magnetometer. Since the robot shell has a radius of 3 cm, this is approximately the closest location the object can be. And as the distance increases, the magnitude of the distortion field reduces. It is noticed that the relation between the magnitude and the distance to the object shows similar trend regardless of the approaching angle. This indicates if the object geometry is known, the distance between the object and robot can be simply calculated from the magnitude. When data in Figure 16 are used for the curve fitting, a simple relation between the magnitude and distance can be approximated in a close form solution as (6):

(6) $$\left|B\right|=D^{-3}\times 5200$$

**Figure 17:**
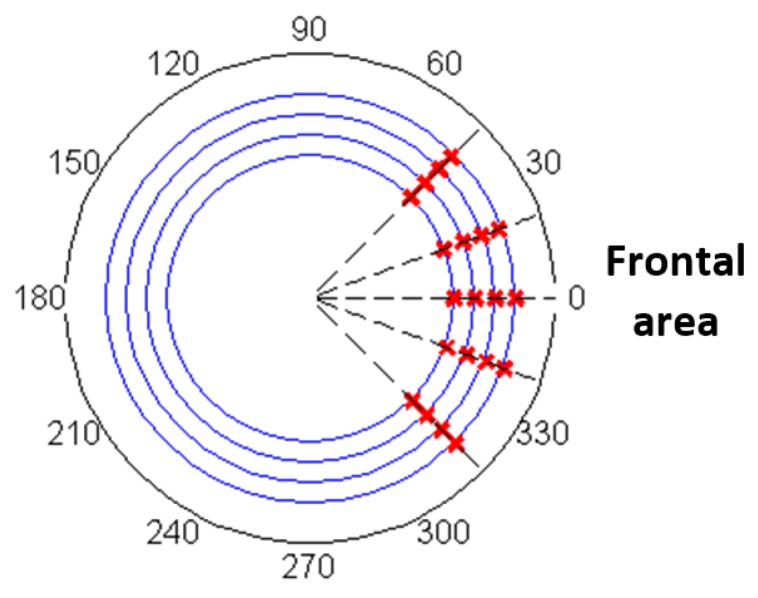
Reconstructed locations of the ferromagnetic objects.

Using this relation, the reconstructed locations of the object are computed and shown in Figure 17, where blue lines indicate the distance to the object to be 3–6 cm and the black dash lines indicate the approaching angles to (−45°, −20°, 0°, 20°, 45°) counterclockwise. The intersection points of the blue line and black dash lines are the actual locations of the obstacle. The red cross represents the reconstructed locations and they correspond to the actual locations accurately.

Since the scope of this paper is obstacle detection and it is reasonable to assume the object geometry is unknown, potential distance detection is not applied in the actual experiments. However, this can be particularly useful when multiple robots are moving together and need to detect the accurate locations of each other.

### 4.2. Obstacle Avoidance Performance

**Figure 18:**
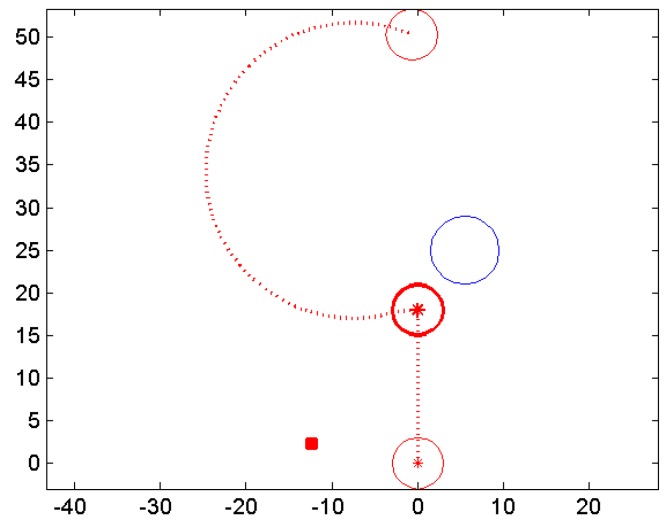
Simulated avoiding course when the robot detects an obstacle.

To validate the proposed obstacle avoidance algorithm, the numerical computing software MATLAB is used to simulate the reaction when one robot detects the presence of an obstacle while in motion. Considering the magnetic noise to be smaller than 10 µT, the actual detection range of the sensor is approximately 3 cm outside the shell based on Figure 16. A waypoint zone is set so that the robot stops moving when it comes close enough to the target waypoint. Simulation parameters are listed in Table 4 and the computed course is shown in Figure 18. The robot path is indicated by the red dot line. When the distance between the robot and the obstacle is smaller than the detection range (the robot detects the obstacle), where the robot location is marked with bold red circle, an intermediate waypoint marked with red rectangular is generated. And after turning 90° counterclockwise, the robot adjusts its course towards the intermediate waypoint. When the distance between the robot and the obstacle increases to a safe level, the robot again adjusts its course towards the original target waypoint. 

**Figure 19:**
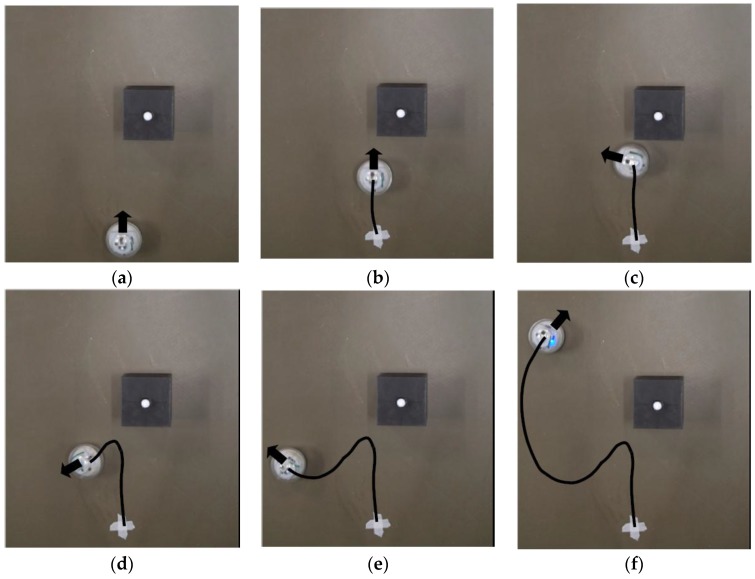
Snapshots of the robot course as it detects and avoids the obstacle while still reaching its target waypoint: (**a**) Robot starts moving; (**b**) Robot moves closer to the obstacle and detects its presence; (**c**) Robot turns counterclosewise after detecting the obstacle; (**d**) Robot adjusts its course towards the intermediate waypoint; (**e**) Robot adjusts the course again to reach its original target waypoint; (**f**) Robot approaches the target waypoint.

**Figure 20:**
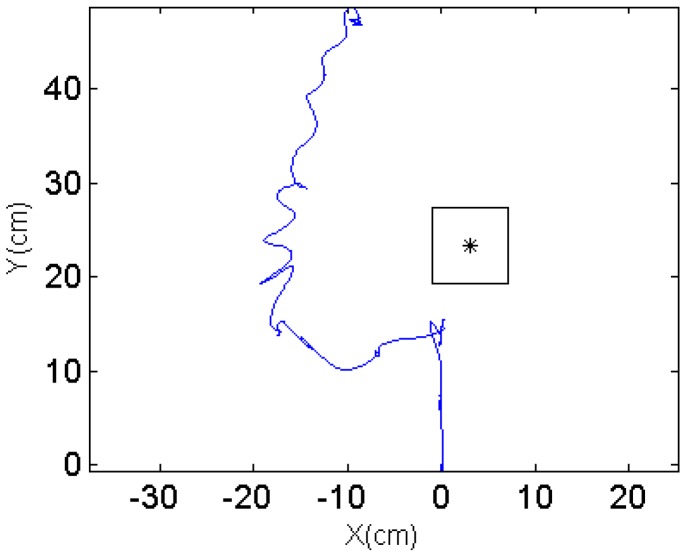
Trajectory from the VICON system.

To validate the robot performance and demonstrate its robustness, two different scenarios are designed so that the obstacle is on different sides when detected by the robot. The robot is expected to automatically choose the turning direction and compute the intermediate waypoint. The snapshots for scenario 1 are shown in Figure 19 and the trajectory captured by the optical motion tracking system is shown in Figure 20.

**Figure 21:**
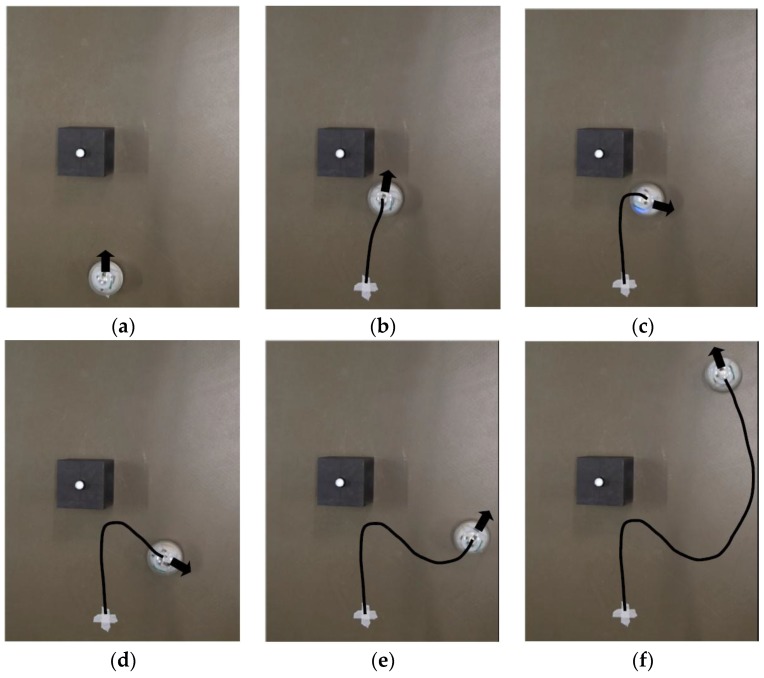
Snapshots of the robot course as it detects and avoid the obstacle while still reaching its target waypoint: (**a**) Robot starts moving; (**b**) Robot moves closer to the obstacle and detects its presence; (**c**) Robot turns closewise after detecting the obstacle; (**d**) Robot adjusts its course towards the intermediate waypoint; (**e**) Robot adjusts the course again to reach its original target waypoint; (**f**) Robot approaches the target waypoint.

**Figure 22:**
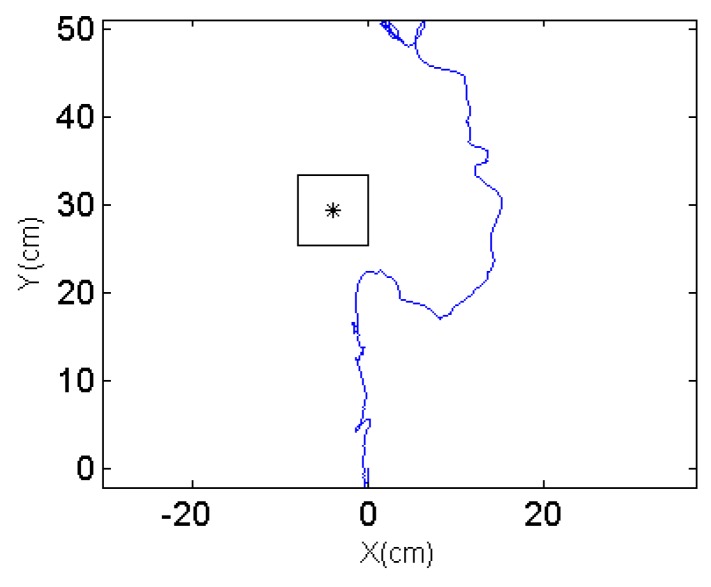
Trajectory from the VICON system.

It can be seen in Figure 19c–e that the robot turns to the left side when detecting an obstacle at its right front. The oscillations along the path in Figure 20 are due to the pitch and roll motion of the robot. The second scenario when the robot detects an obstacle at its left front is also captured using VICON system and the trajectories are shown in Figure 21 and Figure 22. 

When the robot meets the obstacle and detects that the obstacle is at its left frontal area, it turns clockwise and proceeds to an intermediate waypoint before resuming to the original target waypoint. It is noted that the trajectories shown in Figure 20 and Figure 22 are not smooth due to the sharp turns in front of the obstacles. To prevent the robot from spinning out of control, the actual control commands executed here have been to stop the robot first and then turn on the spot. In the conducted experiments, the robot is capable of avoiding the obstacle without breakdown in the normal rolling behavior. The stability of the robot during the stop and turn action depends on the center of mass, motor torque, robot moving speed and so on. These issues are outside the scope of this manuscript and will not be discussed in detail.

## 5. Conclusions

In this paper, a passive magnetic field based proximity sensor has been implemented on a VIRGO miniature spherical robot and obstacle avoidance behavior has been achieved. It has been demonstrated that the improved robot can avoid the obstacles before physical contact and still reach its designed target waypoint. Implementation of this novel sensing system does not require reconfiguration of the robot and there is no physical change in the dimensions of this compact spherical robot. However, due to the inclusion of the magnets, the center of gravity of the robot has been shifted, causing the dynamic performance of the robot to change slightly. This issue can be addressed through tuning the motion controller. With the detailed design optimization strategies, the proximity sensor can also be developed for other platforms. When extending the research scope to a multiple robot scenario, the proposed method can be beneficial for multi-robot navigation, interaction and formation. 

## Figures and Tables

**Table 1:** Parameters of Four Magnets with Different Aspect Ratio.

	*V* = 1.024 cm^3^		*V* = 2.048 cm^3^	
	Magnet *a*	Magnet *b*	Magnet *c*	Magnet *d*
*L* (cm)	0.635	1.27	0.635	1.27
*H* (cm)	0.635	0.1588	1.27	0.3175
γ	1	8	1/2	4
Surface Field (T)	0.5754	0.1448	0.6353	0.2704

**Table 2:** Hardware Parameters of the Robot.

**Motor**	Faulhaber 1512U003SR 112:1, Max RPM: 119
**Wheel**	Diameter: 10 mm, Width: 2 mm, Track width: 30 mm
**Controller**	STM32F411RE (ARM Cortex M4, 100 MHz, 128 Kb RAM, 512 Kb Flash)
**IMU**	Bosch BNO055
**Magnets**	Length: 1/2 inch, Height: 1/8 inch, Grade: N52
**Magnetometer**	MAG3110

**Table 3:** Detected Approaching Angles Compared with Designed Angles.

Frontal Area					
Designed angle (°)	−45	−20	0	20	45
Average detected angle (°)	−46.8	−18.2	2.2	22.7	46.2
Average error (%)	1.9	1.5	2.4	2.9	1.4
Maximum detection error (°)	3.1	2.2	3.2	2.9	2.3
Maximum error (%)	3.4	2.4	3.5	3.3	2.5

**Table 4:** Simulation Parameters.

**Robot Radius**	3 cm	Initial location of the robot	(0 cm, 0 cm)
**Detection Range**	3 cm outside the shell	Location of the obstacle	(5 cm, 25 cm)
**Waypoint Zone**	Radius: 1 cm	Target waypoint	(0 cm, 50 cm)

## Appendix A

By transferring the vector field into the real sensor coordinate system (*X-Y-Z*), the distortion field can be expressed as below:

(A1) $$\Delta B=B_{i}=T\times \frac{\mu_{0}}{4\pi }\left(\oint_{S1+S4}\frac{-\sigma_{i}(R-R_{i})}{{\left|R-R_{i}\right|}^{3}}dS+\oint_{S2+S3}\frac{\sigma_{i}(R-R_{i})}{{\left|R-R_{i}\right|}^{3}}dS\right)$$

where $T=\left[\begin{array}{ccc} \cos2\alpha & -\sin2\alpha & 0\\ \sin2\alpha & \cos2\alpha & 0\\ 0 & 0 & 1 \end{array}\right]$, accounting for the transformation matrix between the real coordinate system and image coordinate system; $\sigma_{i}$ represents the image magnetic charge, which is a constant value when assuming the magnetization of the magnet is constant; **R** and $R_{i}$ represent the location of the field and image charges in the *x-y-z* coordinate system, S2, S3 and S4 refers to the pole areas of the image magnets.

By rearranging the integration operator, (A1) can be reformed as below:

(A2) $$\begin{array}{l} \Delta B=T\times \frac{\mu_{0}}{4\pi }(\oint_{S1+S4}\frac{-\sigma_{i}R}{{\left|R-R_{i}\right|}^{3}}dS+\oint_{S2+S3}\frac{\sigma_{i}R}{{\left|R-R_{i}\right|}^{3}}dS\\ \begin{array}{c} + \end{array}\oint_{S1+S4}\frac{\sigma_{i}R_{i}}{{\left|R-R_{i}\right|}^{3}}dS+\oint_{S2+S3}\frac{-\sigma_{i}R_{i}}{{\left|R-R_{i}\right|}^{3}}dS) \end{array}$$

Due to the symmetric structure, the last two terms vanishes when the sensor is located at the origin of the real coordinate system. The Equation (A2) can be further simplified as below:

(A3) $$\Delta B=\frac{\mu_{0}\sigma_{i}(\oint_{S2+S3}\frac{1}{{\left|R-R_{i}\right|}^{3}}dS-\oint_{S1+S4}\frac{1}{{\left|R-R_{i}\right|}^{3}}dS)}{4\pi }(T\times R)$$

Considering **T** refers to the transformation matrix from the *x-y-z* system to the *X-Y-Z* system and **R** refers to the location of the field in the *x-y-z* coordinate system, the term T×R represents the vector pointing from the center of the image magnets to the field location, expressed in the *X-Y-Z* coordinate system. The center of the image magnets can be expressed in the *X-Y-Z* coordinate system as ${\left[\begin{array}{ccc} 2D\cos\alpha \cos\alpha & 2D\cos\alpha \sin\alpha & 0 \end{array}\right]}^{T}$. Then the normalized field variation can be expressed as below:

(A4) $$\frac{\Delta B}{\left|\Delta B\right|}=\frac{R_{S}}{\left|R_{S}\right|}-\left[\begin{array}{c} \cos\alpha \\ \sin\alpha \\ 0 \end{array}\right]$$

where $R_{S}$ represents the field location in the *X-Y-Z* coordinate system. Then, when $R_{S}={\left[\begin{array}{ccc} 0 & 0 & 0 \end{array}\right]}^{T}$, the approaching angle can be easily computed from the normalized field variation as below:

(A5) $$\alpha =\arctan(\frac{\Delta B_{y}}{\Delta B_{x}})\;\mathrm{when}\;R_{S}={\left[\begin{array}{ccc} 0 & 0 & 0 \end{array}\right]}^{T}$$

## References

[1] Pol R.S., Murugan M. A review on indoor human aware autonomous mobile robot navigation through a dynamic environment survey of different path planning algorithm and methods. Proceedings of the International Conference on Industrial Instrumentation and Control (ICIC).

[2] Volos C.K., Kyprianidis I.M., Stouboulos I.N. (2013). Experimental investigation on coverage performance of a chaotic autonomous mobile robot. Robot. Auton. Syst..

[3] Martinez D., Teixidó M., Font D., Moreno J., Tresanchez M., Marco S., Palacín J. (2014). Ambient intelligence application based on environmental measurements performed with an assistant mobile robot. Sensors.

[4] Sánchez-Hermosilla J., González R., Rodríguez F., Donaire J. (2013). Mechatronic description of a laser autoguided vehicle for greenhouse operations. Sensors.

[5] Barai S., Dey A., Sau B. Path following of autonomous mobile robot using passive rfid tags. Proceedings of the 2016 International Conference on Microelectronics, Computing and Communications (MicroCom).

[6] Zhang J., Jiang Y., Wang K. A modified fastslam for an autonomous mobile robot. Proceedings of the 2016 IEEE International Conference on Mechatronics and Automation.

[7] Zhao R., Lee D.H., Li T.T., Lee H.K. Autonomous navigation of a mobile robot in unknown environment based on fuzzy inference. Proceedings of the 2015 International Automatic Control Conference (CACS).

[8] Boukattaya M., Jallouli M., Damak T. (2012). On trajectory tracking control for nonholonomic mobile manipulators with dynamic uncertainties and external torque disturbances. Robot. Auton. Syst..

[9] Roozegar M., Mahjoob M.J., Jahromi M. (2016). Optimal motion planning and control of a nonholonomic spherical robot using dynamic programming approach: Simulation and experimental results. Mechatronics.

[10] Armour R.H., Vincent J.F.V. (2006). Rolling in nature and robotics: A review. J. Bionic Eng..

[11] Chase R., Pandya A. (2012). A review of active mechanical driving principles of spherical robots. Robotics.

[12] Zhu Y., Jin B., Wu Y., Guo T., Zhao X. (2016). Trajectory correction and locomotion analysis of a hexapod walking robot with semi-round rigid feet. Sensors.

[13] Shi L., Guo S., Li M., Mao S., Xiao N., Gao B., Song Z., Asaka K. (2012). A novel soft biomimetic microrobot with two motion attitudes. Sensors.

[14] Seeman M., Broxvall M., Saffiotti A., Wide P. An autonomous spherical robot for security tasks. Proceedings of the 2006 IEEE International Conference on Computational Intelligence for Homeland Security and Personal Safety.

[15] Lin X., Guo S., Tanaka K., Hata S. Underwater experiments of a water-jet-based spherical underwater robot. Proceedings of the 2011 IEEE International Conference on Mechatronics and Automation.

[16] Pan S., Shi L., Guo S. (2015). A kinect-based real-time compressive tracking prototype system for amphibious spherical robots. Sensors.

[17] Ajay V.A., Suherlan A.P., Soh G.S., Foong S., Wood K., Otto K. (2015). Localization and trajectory tracking of an autonomous spherical rolling robot using imu and odometry. Proceedings of the ASME 2015 International Design Engineering Technical Conferences and Computers and Information in Engineering Conference.

[18] Wu F., Maréchal L., Vibhute A., Foong S., Soh G.S., Wood K.L. A compact magnetic directional proximity sensor for spherical robots. Proceedings of the 2016 IEEE International Conference on Advanced Intelligent Mechatronics (AIM).

[19] Lau D., Eden J., Oetomo D. (2015). Fluid motion planner for nonholonomic 3-D mobile robots with kinematic constraints. IEEE Trans. Robot..

[20] Palmieri L., Koenig S., Arras K.O. Rrt-based nonholonomic motion planning using any-angle path biasing. Proceedings of the 2016 IEEE International Conference on Robotics and Automation (ICRA).

[21] Karaman S., Frazzoli E. Sampling-based optimal motion planning for non-holonomic dynamical systems. Proceedings of the 2013 IEEE International Conference on Robotics and Automation.

[22] Strakowski M.R., Kosmowski B.B., Kowalik R., Wierzba P. (2006). An ultrasonic obstacle detector based on phase beamforming principles. IEEE Sens. J..

[23] Mu W.-Y., Zhang G.-P., Huang Y.-M., Yang X.-G., Liu H.-Y., Yan W. (2016). Omni-directional scanning localization method of a mobile robot based on ultrasonic sensors. Sensors.

[24] Ko N., Kuc T.-Y. (2015). Fusing range measurements from ultrasonic beacons and a laser range finder for localization of a mobile robot. Sensors.

[25] Mori T., Scherer S. First results in detecting and avoiding frontal obstacles from a monocular camera for micro unmanned aerial vehicles. Proceedings of the 2013 IEEE International Conference on Robotics and Automation.

[26] Bengochea-Guevara J., Conesa-Muñoz J., Andújar D., Ribeiro A. (2016). Merge fuzzy visual servoing and GPS-based planning to obtain a proper navigation behavior for a small crop-inspection robot. Sensors.

[27] Hernández A., Gómez C., Crespo J., Barber R. (2016). Object detection applied to indoor environments for mobile robot navigation. Sensors.

[28] Herrero D., Martínez H. (2011). Fuzzy mobile-robot positioning in intelligent spaces using wireless sensor networks. Sensors.

[29] Segura M., Auat Cheein F., Toibero J., Mut V., Carelli R. (2011). Ultra wide-band localization and slam: A comparative study for mobile robot navigation. Sensors.

[30] Scalise L., Primiani V.M., Russo P., Shahu D., Mattia V.D., Leo A.D., Cerri G. (2012). Experimental investigation of electromagnetic obstacle detection for visually impaired users: A comparison with ultrasonic sensing. IEEE Trans. Instrum. Meas..

[31] Lee K.-M., Li M. Magnetic field localization method for guiding visually impaired applications. Proceedings of the 2013 IEEE/ASME International Conference on Advanced Intelligent Mechatronics.

[32] Nara T., Suzuki S., Ando S. (2006). A closed-form formula for magnetic dipole localization by measurement of its magnetic field and spatial gradients. IEEE Trans Magn..

[33] Lee K.M., Li M., Lin C.Y. A novel way-finding method based on geomagnetic field effects and magnetic tensor measurements for visually impaired users. Proceedings of the 2015 IEEE International Conference on Advanced Intelligent Mechatronics (AIM).

[34] The American Practical Navigator (Nima 2002 pdf Edition) 2013. https://en.wikisource.org/wiki/The_American_Practical_Navigator/Chapter_6.

[35] Minguez J., Montano L., Santos-Victor J. Reactive navigation for non-holonomic robots using the EGO-kinematic space. Proceedings of the IEEE International Conference on Robotics and Automation 2002.

[36] Petti S., Fraichard T. Safe motion planning in dynamic environments. Proceedings of the 2005 IEEE/RSJ International Conference on Intelligent Robots and Systems.

